# Ferroptosis and autophagy induced cell death occur independently after siramesine and lapatinib treatment in breast cancer cells

**DOI:** 10.1371/journal.pone.0182921

**Published:** 2017-08-21

**Authors:** Shumei Ma, Rebecca F. Dielschneider, Elizabeth S. Henson, Wenyan Xiao, Tricia R. Choquette, Anna R. Blankstein, Yongqiang Chen, Spencer B. Gibson

**Affiliations:** 1 Research Institute in Oncology and Hematology, CancerCare Manitoba, University of Manitoba, Winnipeg, Manitoba, Canada; 2 Department of Biochemistry and Medical Genetics, University of Manitoba, Winnipeg, Manitoba, Canada; 3 Key Laboratory of Radiobiology (Ministry of Health), School of Public Health, Jilin University, Changchun, Jilin, China; Swedish Neuroscience Institute, UNITED STATES

## Abstract

Ferroptosis is a cell death pathway characterized by iron-dependent accumulation of reactive oxygen species (ROS) within the cell. The combination of siramesine, a lysosome disruptor, and lapatinib, a dual tyrosine kinase inhibitor, has been shown to synergistically induce cell death in breast cancer cells mediated by ferroptosis. These treatments also induce autophagy but its role in this synergistic cell death is unclear. In this study, we determined that siramesine and lapatinib initially induced ferroptosis but changes to an autophagy induced cell death after 24 hours. Furthermore, we found that intracellular iron level increased in a time dependent manner following treatment accompanied by an increase in ROS. Using the iron chelator deferoxamine (DFO) or the ROS scavenger alpha-tocopherol decreased both autophagy flux and cell death. We further discovered that decreased expression of the iron storage protein, ferritin was partially dependent upon autophagy degradation. In contrast, the expression of transferrin, which is responsible for the transport of iron into cells, is increased following treatment with lapatinib alone or in combination with siramesine. This indicates that ferroptosis and autophagy induced cell death occur independently but both are mediated by iron dependent ROS generation in breast cancer cells.

## Introduction

Ferroptosis is a new form of programmed cell death characterized by iron dependent increased in reactive oxygen species (ROS) [1]. Inhibiting the cystine-glutamate antiporter (system X_c_^−^) causes the depletion of glutathione (GSH), the major cellular antioxidant [1]. This leads to ferroptosis through the loss of cellular redox homeostasis. In addition, alterations in iron transport proteins increases iron mediated ROS that also leads to ferroptosis [2]. This illustrates the central role ROS plays in regulating ferroptosis.

Autophagy an intracellular catabolic process involving lysosomes that could lead to programmed cell death through extensive degradation of intracellular structures or organelles [3]. Autophagy is usually characterized by the formation of double membranes called autophagosomes. These autophagosomes fuse with lysosomes forming autolysosomes where degradation occurs [4]. Similar to ferroptosis, this process is regulated by ROS levels as increased oxidative stress leads to autophagy induce cell death. In recent reports, autophagy contributes to ferroptosis through degradation of the iron-storage protein, ferritin [5]. Ferritin is a universal intracellular protein that stores iron and releases it in a controlled fashion. Degradation of ferritin cause increased iron levels leading to accumulation of ROS in cells ultimately leading to cell death. Whether ferroptosis and autophagy induced cell death are dependent upon each other is currently not well understood.

We have found that siramesine disrupts lysosome membranes leading to cell death and in combination with lapatinib (a tyrosine kinase inhibitor of ErbB1 and ErbB2) induces ferroptosis in breast cancer cells [6]. This occurs through inhibiting the iron transport system leading to an increased in ROS and cell death. In addition, both siramesine and lapatinib induce autophagy [7, 8] but the role of autophagy in siramesine and lapatinib induced synergistic cell death is unknown. In this study, we investigated the role of ferroptosis and autophagy on siramesine and lapatinib induced cell death and the role of intracellular iron and ROS plays in regulating both ferroptosis and autophagy induced cell death in breast cancer cells.

## Results

### Siramesine and lapatinib induced ferroptosis and autophagic cell death at different times

**Fig 1:**
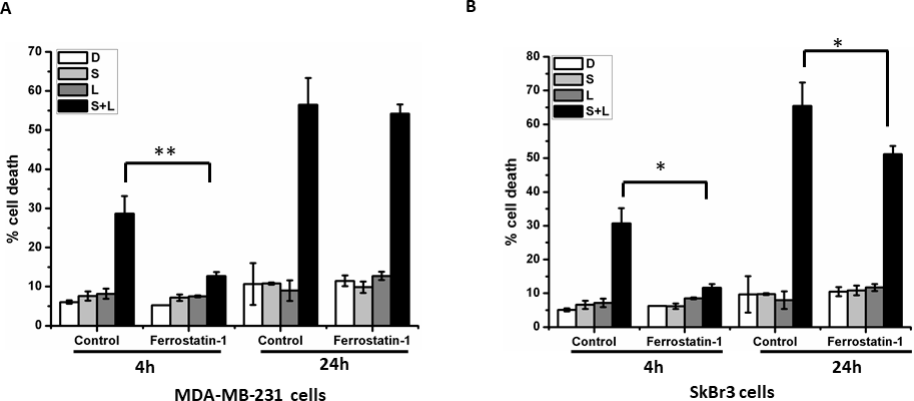
Siramesine and lapatinib induced ferropoptosis at 4h and autophagic cell death at 24 hours. (A, B) The effects of ferrostain-1 (5 microM) on cell death under siramesine and lapatinib treatment in MDA MB 231 and SKBR3 for 4 and 24 hours respectively. Cell death was quantified by flow cytometer. Before treatment under siramesine and lapatinib, cells were pretreated with ferrostain-1 for 1 hour. These results were representative of three independent experiments (n = 3). *p<0.05; **p<0.01.

To determine whether the extent of ferroptosis, apoptosis or autophagy induced cell death following siramesine and lapatinib treatment, we pretreated MDA MB 231 and SKBR3 cells with ferrostatin-1 (Fer-1, ferroptosis inhibitor), 3-MA (autophagy inhibitor) or Z-VAD (apoptosis inhibitor) and determined the amount of cell death. We found that Fer-1 decreased siramesine and lapatinib induce cell death from 30% to 12% at 4 hours in MDA MB 231 cells (Fig 1), and from 30% to 11% at 4 hours and from 65% to 50% at 24 hours in SkBr3 cells (Fig 1). This was further confirmed in MCF-7 cells (S1 Fig). In addition, the sequence of siramsine and lapatinib treatment failed to effect the increase of cell death (S2 Fig). The amount of apoptosis as measured by sub-G1 peak analysis failed to increase after siramesine and lapatinib treatment and z-VAD pretreatment failed to further decrease cell death (S3 Fig). This indicates that both ferroptosis and autophagy contribute to siramesine and lapatinib induced cell death.

**Fig 2:**
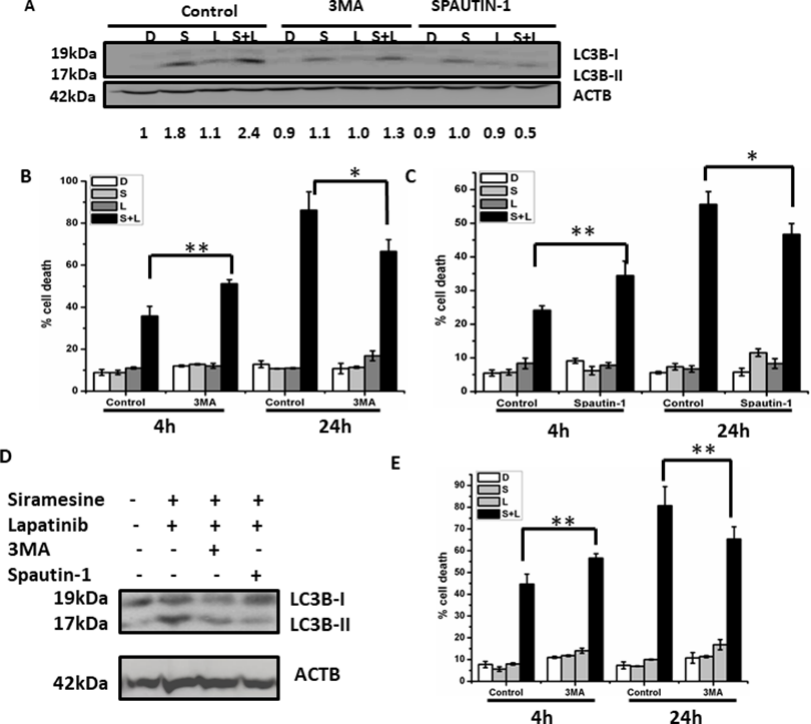
Autophagy inhibitors increase cell death at an early time of siramesine and lapatinib treatment but inhibit cell death at a later time. **(A)** Inhibition of autophagy by autophagy inhibitors 3-methyladenine (3-MA, 2 mM, and same hereafter) and spautin-1 (3 microM, and same hereafter) under siramesine and lapatinib treatment as demonstrated by LC3-II western blot in MDA MB 231 cells. **(B)** The effects of 3-MA on cell death under siramesine and lapatinib treatment in MDA MB 231 cells for 4 and 24 hours. **(C)** The effects of spautin-1 on cell death under siramesine and lapatinib treatment in MDA MB 231 for 4 and 24 hours. Cell death was quantified by flow cytometry as described in the Materials and Methods section (and same hereafter). Before treatment with siramesine and lapatinib, cells were pretreated with 3-MA or spautin-1 for 1 hour (and same hereafter). (D) Inhibition of autophagy by autophagy inhibitors 3-methyladenine (3-MA, 2 mM) and spautin-1 (3 microM) under siramesine and lapatinib treatment as demonstrated by LC3-II western blot in SKBR3 cells. (E) The effects of 3-MA on cell death under siramesine and lapatinib treatment in SKBR3 cells for 4 and 24 hours. These results were representative of three independent experiments (n = 3). *p<0.05; **p<0.01.

**Fig 3:**
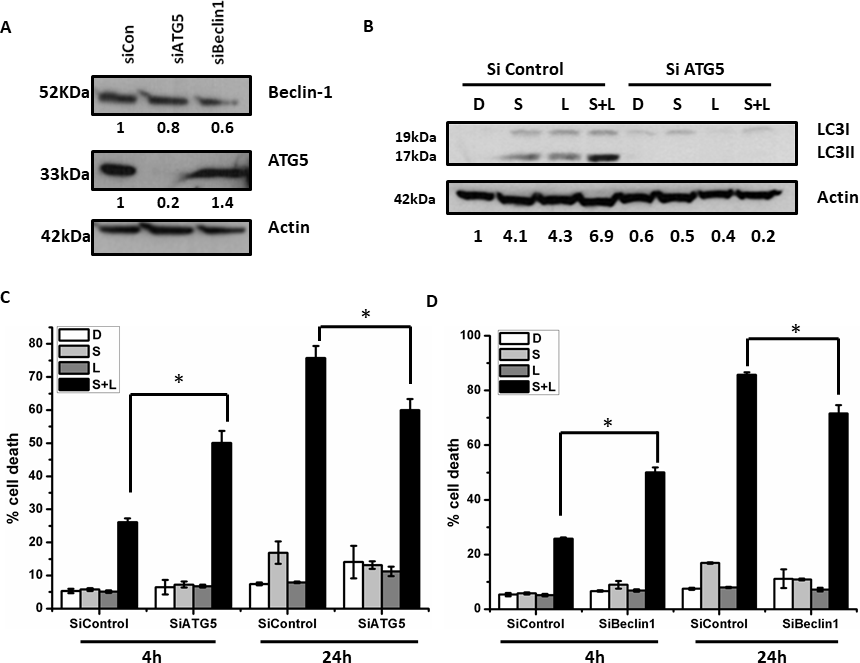
Knockdown of autophagy genes increases cell death during early treatment times with siramesine and lapatinib but inhibits cell death at a later times. (A) Knockdown of autophagy genes *Atg5* and *Becn1* by siRNAs as demonstrated by western blot of Atg5 and Beclin-1, respectively, in MDA MB 231 cells. The protein level of Atg5 was represented by the Atg5-Atg12 complex since the conjugation between these two proteins is an essential step during a functional autophagy process (and same hereafter). (B) Inhibition of autophagy by knockdown of *Atg5* as demonstrated by western blot of LC3I/LC3-II in MDA MB 231 cells. **(**C, D) Effects of knockdown of *Atg5* or *Becn1* on siramesine and lapatinib-induced cell death at 4 and 24 hours in MDA MB 231 cells. These results were representative of three independent experiments (n = 3), *p<0.05.

Autophagy contributes to both survival and cell death [3]. We inhibited autophagy with 3-methyladenine (3-MA) and spautin-1 and determined the amount of siramesine and lapatinib induced autophagy and cell death. We found autophagy was inhibited (Fig 2 and S4 Fig) and cell death was increased from 38% to 52% after 3-MA addition and from 25% to 35% after addition of spautin-1 at 4 hour in MDA MB 231 cells (Fig 2). In contrast at 24 hours, 3-MA inhibited siramesine and lapatinib-induced cell death form 90% to 65% and spautin-1 inhibited siramesine and lapatinib-induced cell death from 87% to 77% (Fig 2). Similar results were found in SkBr3 cells (Fig 2). Furthermore, when the autophagy genes *Atg5* and *Becn1* were knocked down blocking autophagy after siramesine and lapatinib treatment (Fig 3 and S5 Fig), siramesine and lapatinib induced cell death was increased at 4 hour from 25% to 50% in atg5 knockdown cells and from 22% to 48% in Becn1 knockdown cells. At 24 hours, Atg5 knockdown inhibited cell death from 76% to 60% and Becn-1 knockdown inhibited cell death from 87% to 58% in MDA MB 231 cells (Fig 3). This suggests that autophagy initially promotes cell survival but after 24 hours switches to promote cell death.

### Autophagy increases over a 24 hour time course following siramesine and lapatinib treatment

**Fig 4:**
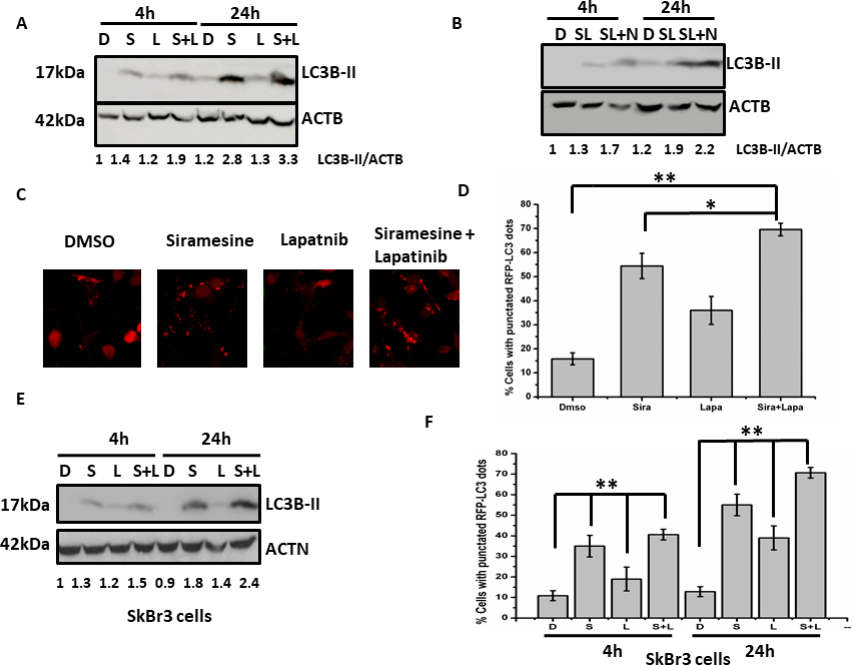
Autophagy flux increases following treatment of cells with siramesine and lapatinib. (A, B) Western blot determination of autophagy protein LC3-II in the absence and presence of NH_4_Cl when MDA MB 231 cells were treated with siramesine and lapatinib for 4and 24 hours. Actin (beta-actin) was used as a loading control (and same hereafter). (C, D) Autophagy was measured by quantifying the number of puncta of a fusion protein of red fluorescent protein LC3 (mRFP-LC3) per cell in MDA MB 231 cells. (E) Western blot determination of autophagy protein LC3-II when SKBR3 cells were treated with siramesine and lapatinib for 4 and 24 hours. (F) Autophagy was measured by quantifying the number of puncta of a fusion protein of red fluorescent protein LC3 (mRFP-LC3) per cell in SKBR3 cells. *p<0.05; **p<0.01.

Both siramesine and lapatinib have been shown to induce autophagy in cancer cells [7, 8]. We determined whether autophagy levels change over a time course when breast cancer cells are treated with both siramesine and lapatinib. The breast cancer cell lines MDA MB 231 were treated with siramesine, lapatinib or in combination over a time course, we measured autophagy by western blotting for the autophagy protein LC3-II, the combination of siramesine and lapatinib gave the largest increase in autophagy (Fig 4). Then, we measured autophagy flux by western blotting for the autophagy protein LC3-II in the absence and presence of the lysosomal inhibitor ammonium chloride (NH4Cl) at 4 and 24 hours following siramesine and lapatinib treatment. The levels of LC3-II, in the presence of NH4Cl, were elevated following siramesine in combination with lapatinib treatment (Fig 4 and S6 Fig), suggesting an increase in autophagy flux. This increase in autophagy was dose dependent with the siramesine or lapatinib treatment alone (S7 Fig). Similar results were observed when autophagy was measured by quantifying the number of puncta of a fusion protein of LC3B (mRFP-LC3B) per cell (Fig 4). Similar results were also found in SkBr3 cells (Fig 4).

### Autophagy promotes ferritin degradation following siramesine and lapatinib treatment

**Fig 5:**
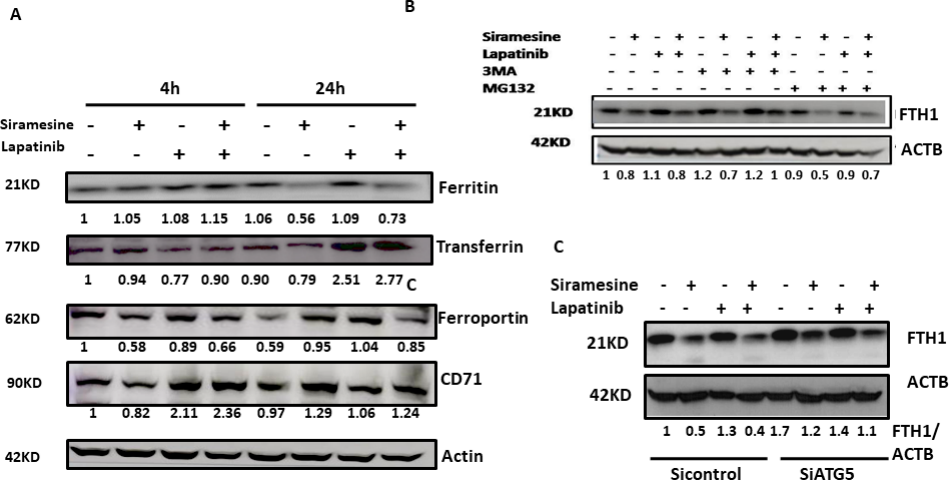
Autophagy promotes ferritin degradation following siramesine and lapatinib treatment in MDA MB 231 cells. (A) MDA MB 231 cells were lysed after treatment with DMSO (D), siramesine (S), lapatinib (L) and siramesine and lapatinib (S + L) for 4 and 24 hour. Western blot determination of iron-related proteins ferritin (FTH1) transferrin, transferrin receptor (CD71), ferroportin (FPN) was performed. Actin was used as a loading control. (B) The effects of 3-MA (2mM) and MG132 (1 microM) on expression level of FTH1 following siramesine and lapatinib treatment in MDA MB 231cells for 24 hours respectively. Before treatment under siramesine and lapatinib, cells were pretreated with 3mM and MG132 for 1hour. (C) Effects of knockdown of *Atg5* on siramesine and lapatinib-induced FTH degradation at 24 hour in MDA MB 231 cells.

Ferritin is the major intracellular iron storage protein complex, which includes FTL1 (ferritin light polypeptide 1) and FTH1 (ferritin heavy polypeptide) [5]. It has been shown increased ferritin expression limits ferroptosis [9]. Furthermore, increased autophagy can degrade ferritin and increase iron level resulting in increased ROS by Fenton reaction [2, 9]. In addition, iron levels are actively regulated in cells through transferrin that transports iron into cells and ferroportin that exports iron out of cells [10]. We investigated whether iron regulatory proteins are altered after siramesine and lapatinib treatment. Cells were treated with siramesine and lapatinib for 4 and 24 hours and lysed and western blotted for ferritin, transferrin, transferrin receptor (CD71) and ferroportin protein. The expression of transferrin was significantly increased after lapatinib alone and in combination with siramesine and lapatinib treatment at 24 hours whereas CD71 was increased at 4 hours following lapatinib alone or in combination with siramesine (Fig 5 and S8 Fig). FTH1 was significantly decreased after siramesine alone or the combination of siramesine and lapatinib at 24 hours but remained unchanged at 4 hours (Fig 5). To determine that degradation of ferritin is by the proteasome or autophagy, autophagy inhibitor 3-MA and proteasome inhibitor MG132 were pretreated for 1 hour then following siramesine and lapatinib treatment, the FTH1 expression was determined. The protein level of FTH1 was significantly increased with 3-MA pretreatment in the siramesine and lapatinib treatment cells but MG132 failed to block Ferritin decreased expression following treatment (Fig 5). Furthermore, when the autophagy gene *Atg5* was knocked down and expression level of FTH1 after siramesine alone or siramesine and lapatinib treatment was increased (Fig 5). In agreement with previous study [6], ferroportin expression decreased only after siramesine and lapatinib treatment but failed to change following autophagy inhibition (Fig 5). These results show alterations in iron transport proteins following siramesine and lapatinib treatment and the decrease in ferritin is due in part to increased autophagy.

### Siramesine and lapatinib increase iron level and ROS generation in a time dependent manner

**Fig 6:**
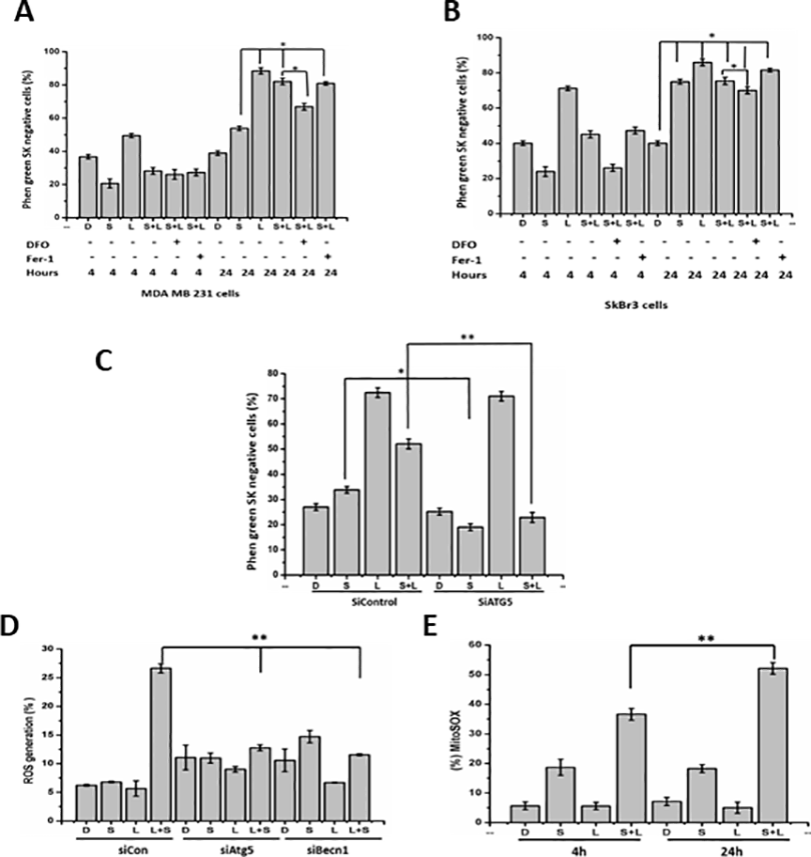
Siramesine and lapatinib increase iron level and ROS generation. (A, B) The effects of DFO (0.5mM) and ferrostain-1 (5 microM) on intracellular chelatable iron under siramesine and lapatinib treatment in MDA MB 231 and SKBR3 cells for 4 and 24 hours respectively. Intracellular chelatable iron was determined using the fluorescent indicator phen green SK, the fluorescence of which is quenched by iron. Samples were examined using a BD FACS Calibur. **(**C) Effects of knockdown of *Atg5* on siramesine and lapatinib-induced intracellular chelatable iron at 24 hour in MDA MB 231 cells. (D) Effects of knockdown of *Atg5* or *Becn1* on siramesine and lapatinib-induced ROS generation at 24 hour in MDA MB 231 cells. (E) The effect of siramesine and lapatinib on mitochondrial ROS generation. Mitochondrial ROS was determined using the fluorescent indicator mitoSOX, samples were examined using a BD FACSCalibur. These results were representative of three independent experiments (n = 3), *p<0.05; **p<0.01.

Our previous studies demonstrated that siramesine and lapatinib induced iron dependent cell death ferroptosis mediated by ROS [6]. We investigated whether iron levels change over a time course following siramesine and lapatinib treatment. We found that iron levels significantly increased at 24 hours in MDA MB 231 and SkBr3 cells after both lapatinib alone or in combination with siramesine (Fig 6). Using the iron chelator DFO, the increase in iron levels was decreased following lapatinib and siramesine treatment, but treating cells with Fer-1 failed to block the increase iron level following treatment (Fig 6) suggesting the increase in iron levels in independent of ferroptosis. Furthermore, when the autophagy genes *Atg5* was knocked down, the iron level following siramesine and lapatinib treatment decreased in MDA MB 231 cells (Fig 6). In contrast, iron levels remained unchanged following lapatinib treatment. When MDA MB 231 cells were treated with siramine and lapating increase ROS levels similar to hydrogen peroxide after 24 hours (S9 and S10 Figs). Furthermore, when the autophagy genes *Atg5* and *Becn1* were knocked down, ROS generation was reduced following siramesine and lapatinib treatment (Fig 6) suggesting autophagy contributes to ROS generation. Similar results were also found using chemical inhibitors of autophagy (S11 Fig). Finally, we found the mitochondrial ROS increased following siramesine alone and was further increased in combination with lapatinib in MDA MB 231 cells (Fig 6). Lapatinib alone failed to increase mitochondrial ROS. This suggests that iron levels are regulation by autophagy following siramesine and lapatinib treatment contributing to increased ROS.

**Fig 7:**
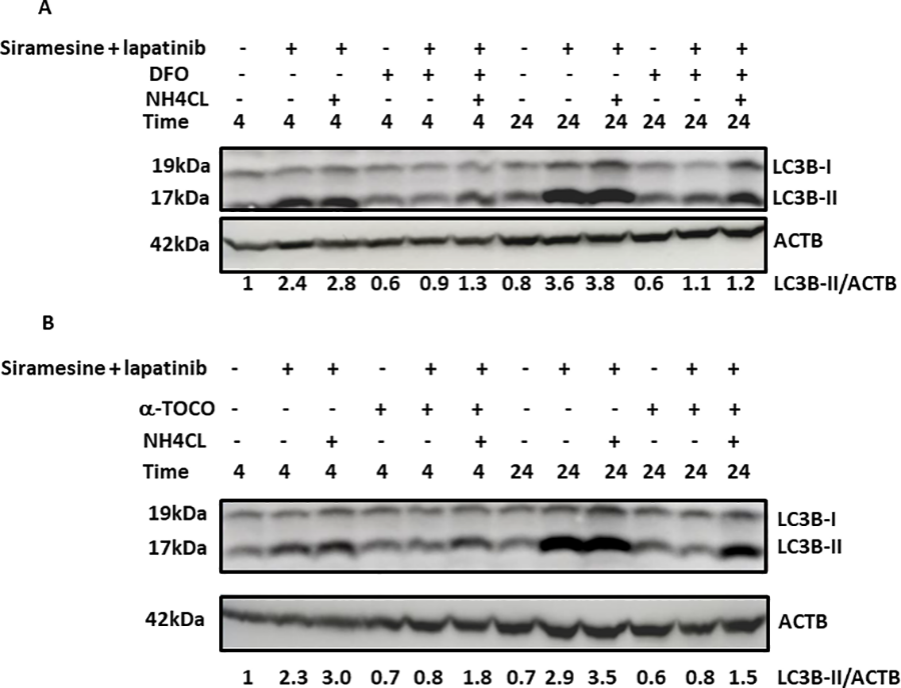
ROS scavenger decreases siramesine and lapatinib induced autophagy. (A, B) The effects of DFO and alpha-tocopherol on autophagic flux determined by the use of NH_4_Cl in the absence and presence of DFO and alpha-tocopherol under siramesine and lapatinib treatment in MDA MB 231 cells for 4 and 24 hours respectively.

**Fig 8:**
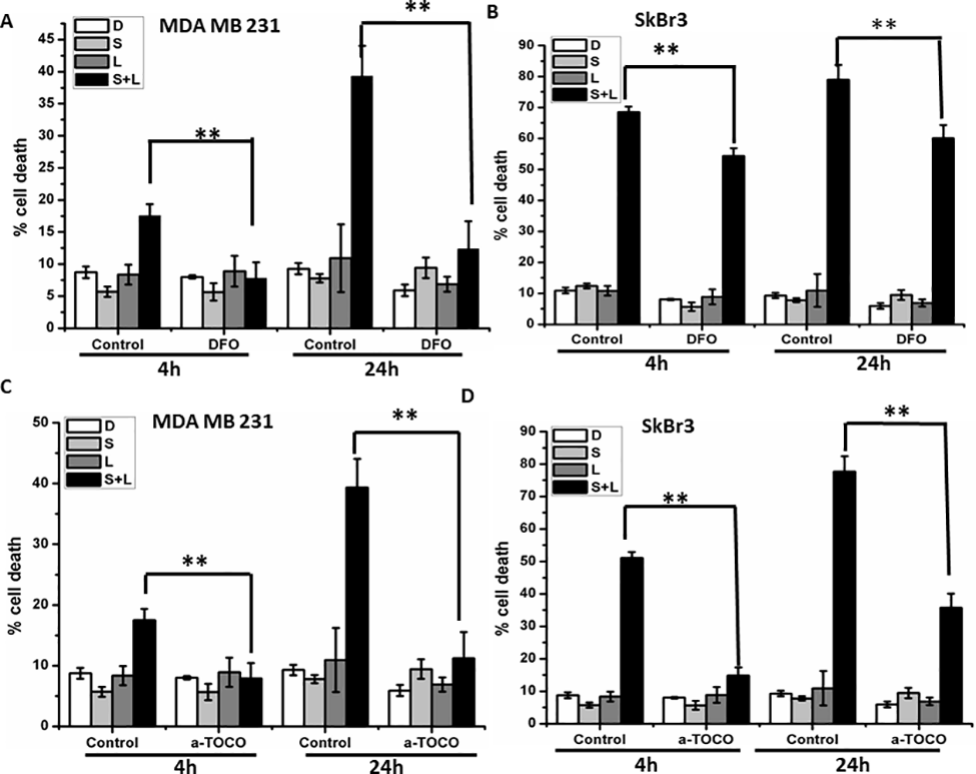
ROS scavenger decreases siramesine and lapatinib induced autophagic cell death. (A, B) The effects of DFO on cell death under siramesine and lapatinib treatment in MDA MB 231 and SKBR3 cells for 4 and 24 hours respectively. **(**C, D) The effects of alpha-tocopherol on cell death under siramesine and lapatinib treatment in MDA MB 231 and SKBR3 cells for 4 and 24 hours respectively. These results were representative of three independent experiments (n = 3), *p<0.05; **p<0.01.

Since ROS generation is increased in a time dependent manner, we investigated the effect of ROS on autophagy and cell death. First, MDA MB 231 cells were pretreated with ROS scavengers DFO or alpha-tocopherol for 1 hour, then treated cells with siramesine and lapatinib for 4 and 24 hours (S12 Fig). We measured autophagy flux by western blotting for the autophagy protein LC3-II in the absence and presence of the NH_4_Cl and found that reducing ROS also reduced autophagy (Fig 7). In addition, DFO and alpha-tocopherol significantly decreased siramesine and lapatinib cell death in both MDA MB 231 and SkBr3 cells (Fig 8). DFO also was effective at blocking lapatinib induced cell death but failed to inhibit siramesine induced cell death suggesting lapatinib induced cell death is mediated by iron induced ROS (S13 Fig). We also found DFO and alpha-tocopherol reduced lipid ROS following siramesine and lapatinib treatment (S14 Fig). When reactive iron (FeCl_3_) was added to cells the amount of autophagy was increased following siramesine and lapatinib treatment (S15 Fig). This indicates that iron induced ROS mediates autophagic cell death.

### Siramesine and lapatinib induced- autophagic cell death is autosis

Recent reports have described a type of autophagic cell death, called autosis that can be induced by treatments with the Tat-BECN1 peptide, starvation and hypoxia–ischemia [11]. Autosis is inhibited by treating cells with Digoxin (Digo) and digitoxigenin (Di) [11]. We investigated whether siramesine and lapatinib induced cell death was due to autosis. When MDA MB 231 cells were treated with siramesine and lapatinib for 24 hours in the absence and presence the autosis inhibitor Di and Digo, autophagic flux induced by siramesine and lapatinib was significantly inhibited (S16 Fig). This corresponded with siramesine and lapatinib induced cell death being significantly inhibited by Di and Digo. In MDA MB 231 cells, Di inhibited cell death induced by siramesine and lapatinib from 59% to 33% whereas Digo inhibited cell death from 55% to 40% after 24 hour of treatment (S16 Fig). Taken together, siramesine and lapatinib induces cell death mainly by ferroptosis initially but switches to autosis at 24 hours.

## Discussion

Previously, we determined that the combination of siramesine, and lapatinib, induced ferroptosis [6]. In this study, we found that siramesine and lapatinib induce both ferroptosis and autophagy induced cell death at different times after treatment. Similarly, we have previously demonstrated that autophagy will initially promote cell survival but elevated and prolonged autophagy will promote cell death under hypoxia [12]. Siramesine and lapatinib treatment increased intracellular iron levels and ROS over 24 hours and ROS scavengers effectively blocked both ferroptosis and autophagy induced cell death. This indicates that different types of cell death can be favored at different times following treatment in breast cancer cells.

Autophagy is an important catabolic process that delivers cytoplasmic material to the lysosome for degradation [4]. It also plays a significant role in regulating iron balance in cells, through the degradation of ferritin [9]. Ferritin is controls the removal of excess iron from the cytoplasm and stores iron in its non-redox active form [5]. Indeed, ferritin has been shown to be degraded in cells by lysosomal acid hydrolases and it is known that ferritin enters lysosomes through the autophagic pathway [9, 13]. Entry of ferritin is also independent of the presence of LAMP-2A, which suggests that ferritin entry does not result from chaperone-mediated autophagy [14]. In agreement with this, we found that LAMP-2A and LC3 fails to co-localized following siramesine and lapatinib treatment indicating siramesine and lapatinib fail to induce chaperone-mediated autophagy (S17 Fig). Our results demonstrate that ferritin degradation is at least partially due to autophagy suggesting it might regulate ROS and cell death following siramesine and lapatinib treatment.

Ferroptosis is dependent upon intracellular iron and prevented by lipophilic antioxidants, such as trolox and vitamin E, and by iron chelators such as deferoxamine but not by well-known small-molecule inhibitors of apoptosis, necrosis or autophagy [1]. The relationship between autophagy and ferroptosis remains unclear. Autophagy could promote ferroptosis by degradation of ferritin, a process known as ferritinophagy [9, 15]. Using RNAi screening, researchers have identified multiple autophagy-related genes as positive regulators of ferroptosis [16]. One of these genes is cargo receptor NCOA4 which is involved in the degradation of ferritin [9, 16]. Consistently, inhibition of ferritinophagy by NCOA4 knockdown blocked the accumulation of cellular labile iron and ROS, as well as eventual blockage of ferroptotic cell death [9, 16]. This suggests ferroptosis is an autophagic cell death process. Conversely, prolonged iron mediated ROS generation can induce autophagy in the absence of ferroptosis. Our results show that ferroptosis can occur independently of autophagy induced cell death. Indeed, inhibition of autophagy at early time points promotes cell death. This indicates ferroptosis and autophagy might cooperate to induce cell death but ferroptosis could be occurring independent of autophagy induced cell death.

Iron is a requisite metal in almost all biological systems. It is required for numerous critical processes such as DNA synthesis, heme and iron-sulfur cluster synthesis [17, 18]. It also plays an important role in the active sites of various enzymes such as cytochrome c, aconitase, and ribonucleotide reductase [17]. However, the levels of iron in the cell need to be tightly balanced, as an excess of iron can have damaging effects due to the generation of ROS [19]. Exogenous application of iron oxide nanoparticles to cells in culture can lead to induction of autophagy due to ROS generation [20]. Addition of reactive iron also leads to increased ROS and autophagy [15]. Our results shown that at 24 hours after treatment iron levels are increased corresponding to increased ROS and autosis. Thus, iron is required ferroptosis but iron mediated ROS could also lead to elevated and prolonged autophagy leading to autosis.

Taken together, our findings provide insight into the regulation of ferroptosis and autosis. This involves altering iron transport and iron storage in a time dependent manner leading to increased ROS and cell death in breast cancer cells. Using clinical agent for the treatment of breast cancer (lapatinib) in combination with a lysosomotropic agent such as siramesine, new therapeutic strategies to overcome apoptotic resistance in breast cancer could be developed.

## Materials and methods

### Reagents and antibodies

Trypan blue solution (Prod. No. T8154), Prussian blue soluble (Prod No.03899), FeCl3•6H2O (Prod No.157740), Deferoxamine (Prod No.D9533), Ferrostatin-1(Prod. No.SML0583), ammonium chloride (NH4Cl) (Prod. No. 254134), MG132 (Prod. No. C2211), N-Acetyl-L-cysteine (Prod No.N7250), and phosphatase inhibitor cocktails 2 &3 (Prod. No. P5726 & P0044) from Sigma-Aldrich (St. Louis, MO, USA), A protease inhibitor cocktail (ref no. 11836 153 001) from Roche Diagnostics (Basel, Switzerland). The siRNAs against Atg5 (sc-41445), Becn1 (sc-29797), and Control siRNA-A (Sc-37007) were purchased from Santa Cruz Biotechnology (Dallas, TX, USA).Primary antibodies: anti-Atg5 (#2630), anti-Beclin-1 (#3738), anti-FTH1(#4393) were purchased from Cell Signaling Technology(Danvers, MA USA), anti-Transferrin (ab9538), anti-SLC40A1(ab85370) from AbCam, and anti-actin from Sigma-Aldrich (Prod. No. A3853). Secondary antibodies: goat anti-rabbit IgG (H+L)-HRP conjugate (Cat. No. 170–6515) and goat anti-mouse IgG (H+L)-HRP conjugate (Cat. No. 170–6516) were obtained from Bio-Rad Laboratories (Mississauga, Ontario, CANADA). MitoSOX Red Mitochondrial Superoxide Indicator (Cat. No. M36008, Phen Green SK, diacetate (Cat. No. P14313) and BODIPY® 581/591 C11 (Lipid Peroxidation Sensor) (Cat. No. D3861) from Life Technologies (Thermo Fisher Scientific, Waltham, MA, USA). Opti-MEM I reduced serum medium (cat: 31985–070) from GIBCO-Life Technologies (Thermo Fisher Scientific).

### Cell culture

The breast cancer cell lines MDA MB-231, SKBR3 were grown in Dulbecco's modified Eagle medium (DMEM, high glucose; GIBCO, cat. 10565–018, Life Technologies) supplemented with 100 units of penicillin per ml plus 100 μg of streptomycin per ml (cat 10378016, Life Technologies) and 10% fetal bovine serum, in a humidified 5% CO2, 37°C incubator. Cells were treated for various times in the absence and presence of a chemical inhibitor.

### Measurement of cell death by flow cytometry

Cell death was measured by staining with trypan blue to detect the plasma membrane integrity through flow cytometry as described previously [21].

### Measurement of mitochondrial ROS by flow cytometry

Mitochondrial ROS generation was determined by flow cytometry with MitoSOX Red Mitochondrial Superoxide Indicator. The samples were collected and stained with 5 mircoM MitoSOX and then were incubated in the dark in a water bath at 37°C for 15 min. The cell suspension was then transferred to a 5 ml FACS tube and analyzed on a flow cytometer within 10 min using Cell Quest software (BD Biosciences, Franklin Lakes, NJ, USA).

### Apoptotic cell death measurement

Flow cytometric analysis of apoptosis was analyzed using both subG1 peak (DNA fragmentation) Cells were stained for subG1 peak analysis after fixation with ethanol using propidium iodide (100 microg/ml). Samples were examined using a BD FACSCalibur.

### Transfection of siRNA and plasmid

The transfection of cells with siRNA was done as described in our previously studies [21].

### Western blot analysis

Cell lysates were collected at the indicated times in 0.1% NP-40 lysis buffer with complete protease inhibitor tablet (Roche, Basel, Switzerland), 1mM phenylmethanesulfonylfluoride (PMSF), and 2 mM sodium orthovanadate (New England BioLabs, Ipswich, MA, USA). Protein levels were quantified with a Pierce BCA kit (Thermo Fisher Scientific) according to the manufacturer’s instructions. Samples were run on 8–10% polyacrylamide gels and transferred onto nitrocellulose membranes (Bio-Rad, Hercules, CA, USA) blocked in 5% milk in TBS-T as per the antibody manufacturer’s suggestions. Secondary antibodies were goat anti-rabbit-HRP or anti-mouse-HRP (Bio-Rad). Detection of protein was with Pierce ECL or Pierce Supersignal Pico (Thermo Fisher Scientific) reagents.

### Iron assay

Intracellular chelatable iron was determined using the fluorescent indicator phen green SK, the fluorescence of which is quenched by iron. Samples were examined using a BD FACSCalibur.

### Statistical analysis

All data were generated with at least three independent experiments. Each experiment in the cell death analysis was carried out by 3–6 replicates. The data were represented as means ± S.D. (n≥3). Student’s t-test was performed for statistical analysis with P>0.05 being considered as statistical significance.

## Supporting information

S1 Fig Siramesine and lapatinib induced more ferroptosis at 4 hours than at 24 hours. The effects of ferrostatin-1 (Fer-1, 5 microM) on cell death after siramesine (S) and lapatinib (L) treatment in Mcf-7 cells for 4 and 24 hours. Cell death was quantified by flow cytometry. Before treatment with siramesine and lapatinib, cells were pretreated with ferrostain-1 for 1 hour. These results are representative of three independent experiments (n = 3). * p<0.05, ** p<0.01.
(TIF)

S2 Fig Synergy observed with siramesine and lapatinib is not dependent on the sequence of treatment. The effects of the sequence of treatment were investigated by treating MDA MB 231 cells with siramesine (S) and lapatinib (L) together, as well as sequentially. Cell death was quantified by flow cytometry after 4 and 24 hours. These results are representative of three independent experiments (n = 3).
(TIF)

S3 Fig Siramesine and lapatinib failed to induce apoptosis in MDA-MB-231 cells. Apoptosis was quantified by flow cytometry by using Sub G1 assay in MDA-MB-231 cells at 4 and 24 hours after treatment with DSMO (D), siramesine (S), lapatinb (L) and siramesine and lapatinib (S + L) in the presence or absence of z-VAD-fmk (10μM). Apoptosis was quantified by flow cytometry by using Sub G1 assay. Error bars represents three independent experiments (n = 3). The data were represented as mean ±S.D.
(TIF)

S4 Fig Autophagy inhibitors reduced LC3II levels. Treatment of MDA-MB 231 cells with DMSO (D), siramesine (S,10 microM) and lapatinib (L, 0.5 microM) or in combination for 24 hours. Cells were also treated with autophagy inhibitors 3MA and Spautin 1 (Sp). The amount of *LC3 I/LC3II* protein expression levels was determined by western blotting. Actin was used as a loading control.
(TIF)

S5 Fig Effect of knockdown of ATG5 and Beclin 1 on siramesine and lapatinib induced autophagy. (A, B) MDA-MB-231 and SKBr3 cells were transfected with control siRNA (siControl) and siRNA against ATG5 and Beclin 1 then treated with siramesine (S,10μM) and lapatinib (L, 0.5μM) or incombinationfor 24 hours. The amount of *LC3 I/LC3II* protein expression levels was determined by western blotting. Actin was used as a loading control.
(TIF)

S6 Fig The extent of autophagy flux following Siramesine + Lapatinib treatment. Treatment of MDA-MB 231 cells for 24 hours with DMSO (D), Siramesine (S), Lapatinib (L), Siramesine + Lapatinib (S+L) alone and in combination with NH_4_Cl and probed for LC3 and Actin.
(TIF)

S7 Fig Dose response for lapatinib and siramesine treatment on autophagic flux. (A, B). MDA MB 231 cells were treated with siramesine at 0, 5, 10, 15, 20 microM in the presence and absence of the lysosomal inhibitor ammonium chloride (NH_4_Cl) (30 mM) for 24 hours respectively. Autophagic flux was quantified by western blot. (B) MDA MB 231 cells were treated with lapatinib at 0, 0.1, 0.25, 0.5, 1.0 and 2.0 microM in the presence and absence of NH4CI for 24 hours respectively. Autophagic flux was quantified by western blot. Actin was used as a loading control.
(TIF)

S8 Fig Expression of iron regulatory proteins following lapatinib treatment for 4 hours in MDA MB 231 cells. MDA MB-231 cells were lysed after treatment with lapatinib at 0, 0.25, 0.5, 1.0 and 2.0 microM. Western blot determination of iron-related proteins ferritin, transferrin, transferrin receptor, FPN was performed.
(TIF)

S9 Fig Siramesine and lapatinib generation of ROS is equivalent to levels generated by H_2_O_2_. The effect of siramesine (S) and lapatinib (L) on mitochondrial ROS generation in MDA MB 231 cells. H_2_O_2_ (100 microM) was used as a positive control for ROS generation. Mitochondrial ROS was determined using the fluorescent indicator mitoSOX, samples were examined using a BD FACSCalibur. These results were representative of three independent experiments (n = 3).
(TIF)

S10 Fig Histogram of siramesine and lapatinib generation of ROS is equivalent to levels generated by H_2_O_2_. The effect of siramesine (S) and lapatinib (L) on mitochondrial ROS generation in MDA MB 231 cells. H_2_O_2_ (100 microM) was used as a positive control for ROS generation. Mitochondrial ROS was determined using the fluorescent indicator mitoSOX (FL3-H), samples were examined using a BD FACSCalibur. These results were representative of three independent experiments (n = 3).
(TIF)

S11 Fig Autophagy inhibitor block siramesine and lapatinib induced ROS generation. MDA MB 231 cells were treated with DMSO (D), siramesine (S), lapatinib (L), and siramesine and lapatinib (S + L) in the presence or absence of autophagy inhibitor 3MA (2mM), bafilomycin A1(10nM), (NH_4_Cl) (10 mM) for 24 hours. ROS level was quantified by DHE using flow cytometer. Error bars represents three independent experiments (n = 3). The data were represented as mean ±S.D *represents statistical significance of p<0.05.
(TIF)

S12 Fig ROS scavenger blocks siramesine and lapatinib induced ROS generation. MDA MB 231 cells were treated with DMSO (D), siramesine (S), lapatinib (L), and siramesine and lapatinib (S + L) in the presence or absence of ROS scavenger DFO(0.2mM), NAC (10mM), a-tocopherol (2 microM) for 24 hours. ROS level was quantified by DHE using flow cytometer. Error bars represents three independent experiments (n = 3). The data were represented as mean ±S.D *represents statistical significance of p<0.05.
(TIF)

S13 Fig DFO block lapatinib induced cell death. MDA MB 231 cells were treated with 10 microM siramesine at 0, 10, 20, 30, 40 microM or with lapatinib at 0, 0.5, 1.0, 2.0 microM in the presence or absence of ROS scavenger DFO (0.2mM) for 24 hours. Total cell death was quantified by flow cytometry by trypan blue exclusion assay. Error bars represents three independent experiments (n = 3). The data were represented as mean ±S.D. * represents statistical significance of p<0.05.
(TIF)

S14 Fig Effect of ROS scavenger on siramesine and lapatinib induced lipid ROS level. MDA MB 231 cells were treated with 10 microM siramesine (S) and 0.5 microM lapatinib (L) in the presence or absence of ROS scavenger DFO (0.2mM), Ferrostatin-1 (Fer-1) (50 microM), alpha-tocopherol (2 microM) for 4 hours, Lipid ROS level was quantified by C11-BIODPY using flow cytometry. Error bars represents three independent experiments (n = 3). The data were represented as mean ±S.D. * represents statistical significance of p<0.05.
(TIF)

S15 Fig Effect of FeCl3 on siramesine and lapatinib induced autophay. Cells were treated with FeCl_3_ (30 microM, pretreated for 3 hours) in combination with siramesine and/or lapatinib. The amount of *LC3 I/LC3II* protein expression levels was determined by western blotting. Actin was used as a loading control.
(TIF)

S16 Fig Siramesine and lapatinib induced autophagic cell death is autosis. **(A)** MDA MB 231 cells were pretreated with the autosis inhibitor digoxin (Di, 5 microM) and digitoxigenin (Digo, 5 mciroM) for 1 hour, then treated with DMSO (D), siramesine (S), lapatinib (L) and siramesine and lapatinib (S + L) in the absence and presence of NH_4_Cl) (30 mM), for 24 hours. Cells were lysed and western blotted for the autophagy protein LC3I/LC3II. Actin was use as a loading control. **(B)** The effects of digoxin and digitoxigenin on cell death under siramesine and lapatinib treatment in MDA MB 231 cells for 24 hours respectively. These results were representative of three independent experiments (n = 3). *p<0.05.
(TIF)

S17 Fig Siramesine and lapatinib failed to induce chaperone-mediated autophagy. MDA-MB-231 cells were transfected with RFP-LC3 then treated with 10 microM siramesine (S) and 0.5 microM lapatinib (L) for 24h. Confocal microscopy does not show the colocolization of LAMP2 (green), which is a lysosome-associated membrane protein type 2A, acts as the receptor for chaperone-mediated autophagy and LC3 (red). LAMP2 was primarily stained with an anti-LAMP2 antibody.
(TIF)

S1 File Copy of PONE-D-17-07275 data 20170714.xls. (XLS)
