# Supplementary figures and images for: Ferroptosis and autophagy induced cell death occur independently after siramesine and lapatinib treatment in breast cancer cells
Source: PLoS One. 2017 Aug 21;12(8):e0182921. doi: 10.1371/journal.pone.0182921 (PMC5565111; doi:10.1371/journal.pone.0182921)

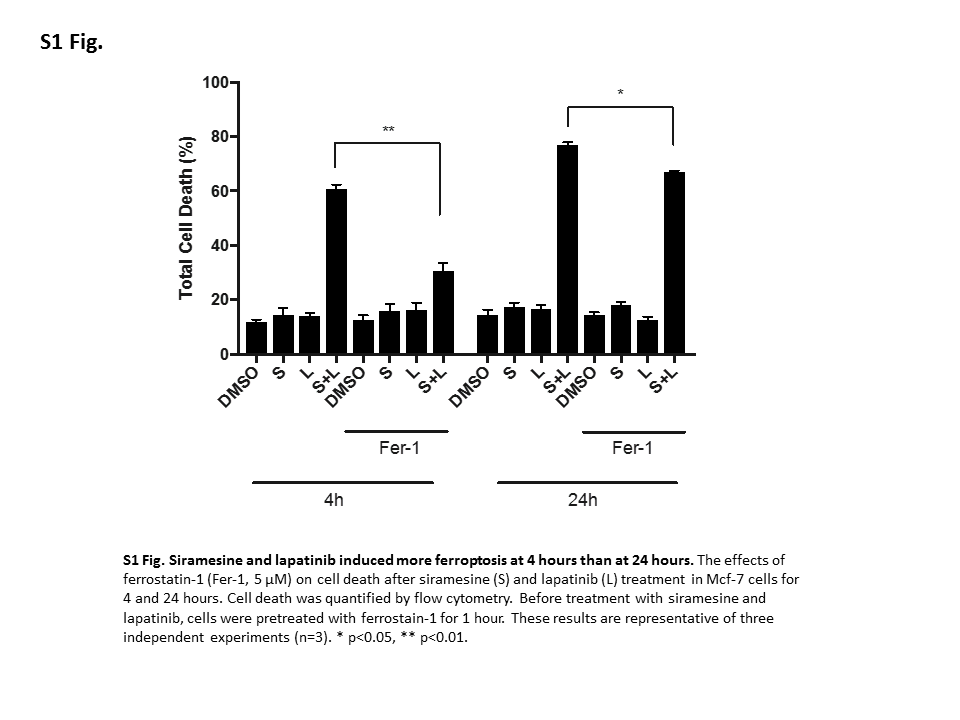

Supplement: S1 Fig — The effects of ferrostatin-1 (Fer-1, 5 microM) on cell death after siramesine (S) and lapatinib (L) treatment in Mcf-7 cells for 4 and 24 hours. Cell death was quantified by flow cytometry. Before treatment with siramesine and lapatinib, cells were pretreated with ferrostain-1 for 1 hour. These results are representative of three independent experiments (n = 3). * p<0.05, ** p<0.01. (TIF) [file pone.0182921.s001.TIF]

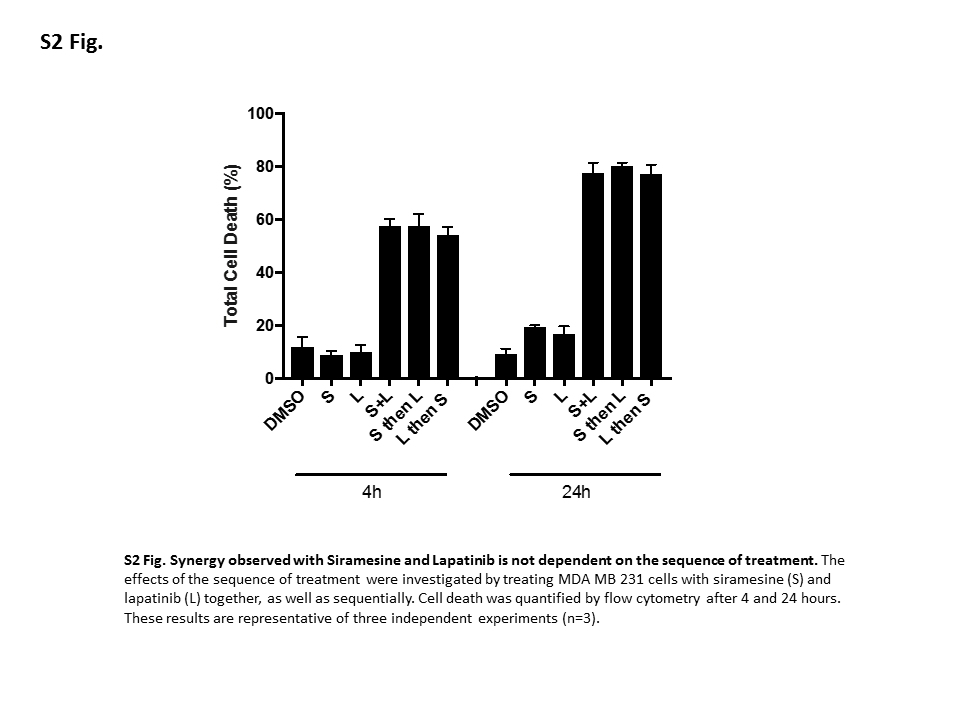

Supplement: S2 Fig — The effects of the sequence of treatment were investigated by treating MDA MB 231 cells with siramesine (S) and lapatinib (L) together, as well as sequentially. Cell death was quantified by flow cytometry after 4 and 24 hours. These results are representative of three independent experiments (n = 3). (TIF) [file pone.0182921.s002.TIF]

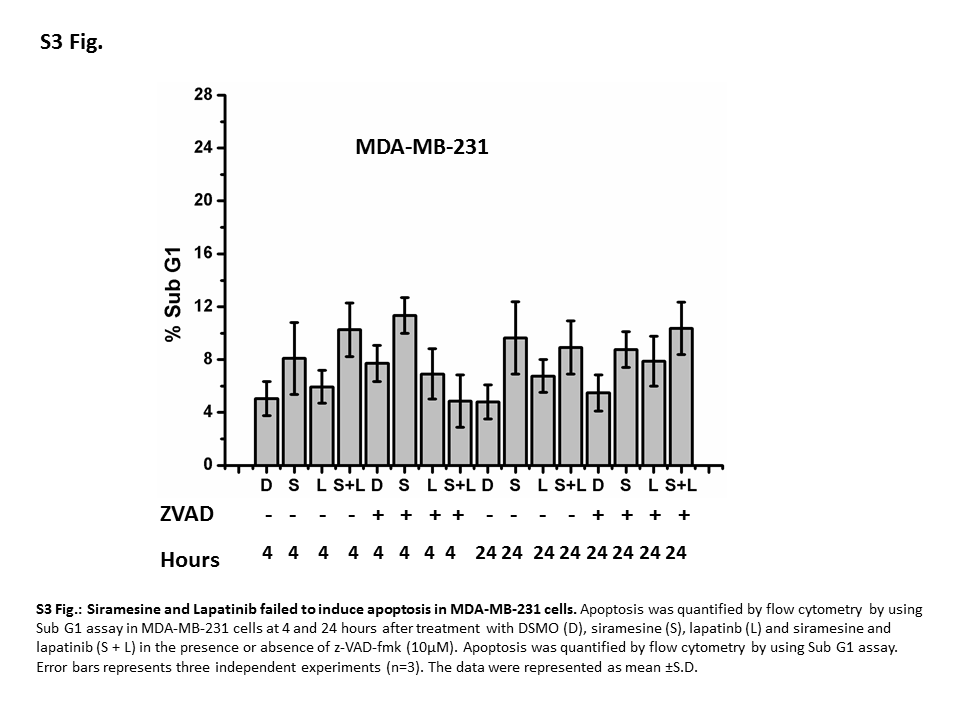

Supplement: S3 Fig — Apoptosis was quantified by flow cytometry by using Sub G1 assay in MDA-MB-231 cells at 4 and 24 hours after treatment with DSMO (D), siramesine (S), lapatinb (L) and siramesine and lapatinib (S + L) in the presence or absence of z-VAD-fmk (10μM). Apoptosis was quantified by flow cytometry by using Sub G1 assay. Error bars represents three independent experiments (n = 3). The data were represented as mean ±S.D. (TIF) [file pone.0182921.s003.TIF]

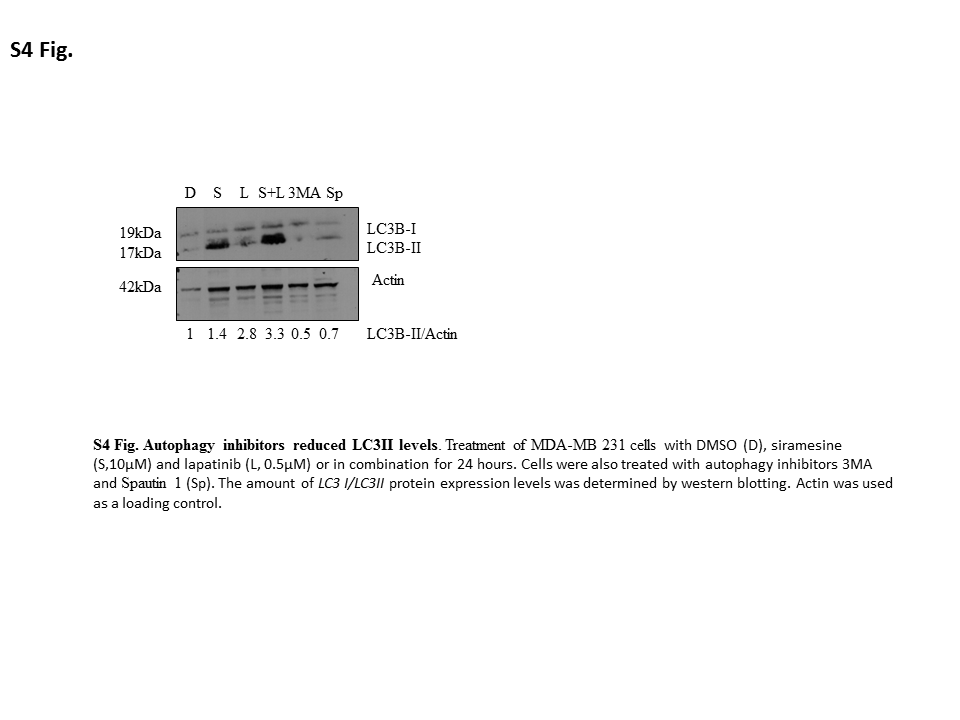

Supplement: S4 Fig — Treatment of MDA-MB 231 cells with DMSO (D), siramesine (S,10 microM) and lapatinib (L, 0.5 microM) or in combination for 24 hours. Cells were also treated with autophagy inhibitors 3MA and Spautin 1 (Sp). The amount of LC3 I/LC3II protein expression levels was determined by western blotting. Actin was used as a loading control. (TIF) [file pone.0182921.s004.TIF]

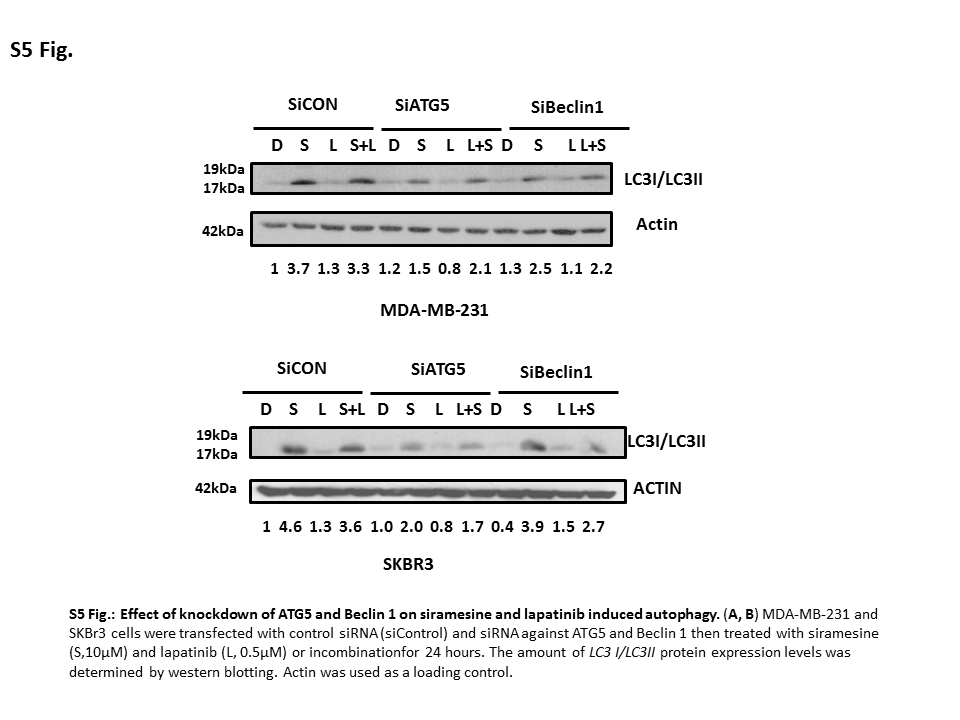

Supplement: S5 Fig — (A, B) MDA-MB-231 and SKBr3 cells were transfected with control siRNA (siControl) and siRNA against ATG5 and Beclin 1 then treated with siramesine (S,10μM) and lapatinib (L, 0.5μM) or incombinationfor 24 hours. The amount of LC3 I/LC3II protein expression levels was determined by western blotting. Actin was used as a loading control. (TIF) [file pone.0182921.s005.TIF]

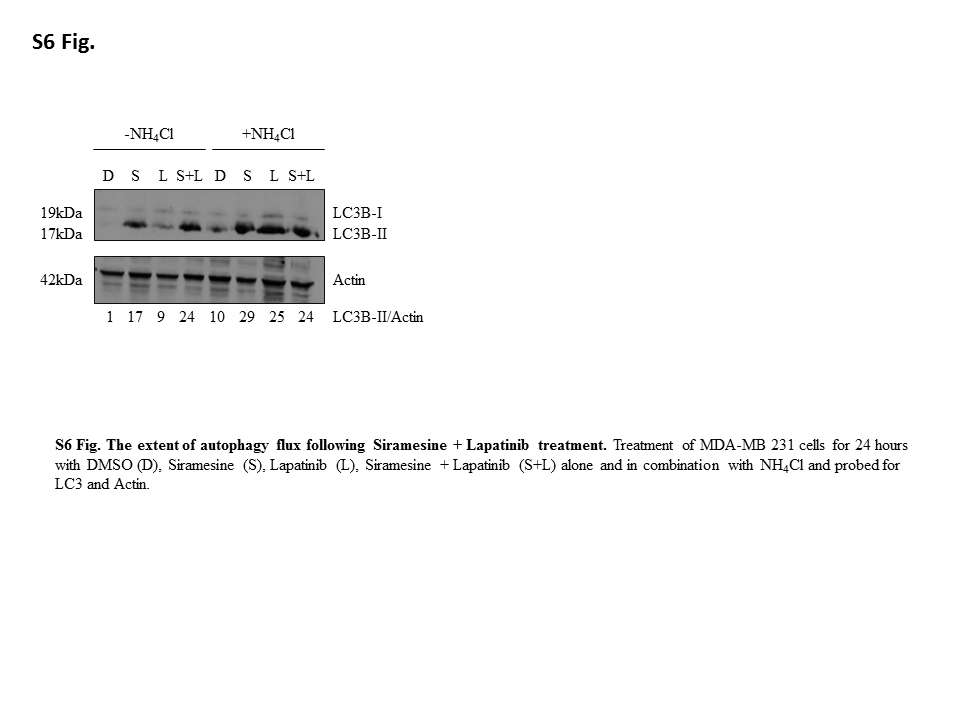

Supplement: S6 Fig — Treatment of MDA-MB 231 cells for 24 hours with DMSO (D), Siramesine (S), Lapatinib (L), Siramesine + Lapatinib (S+L) alone and in combination with NH4Cl and probed for LC3 and Actin. (TIF) [file pone.0182921.s006.TIF]

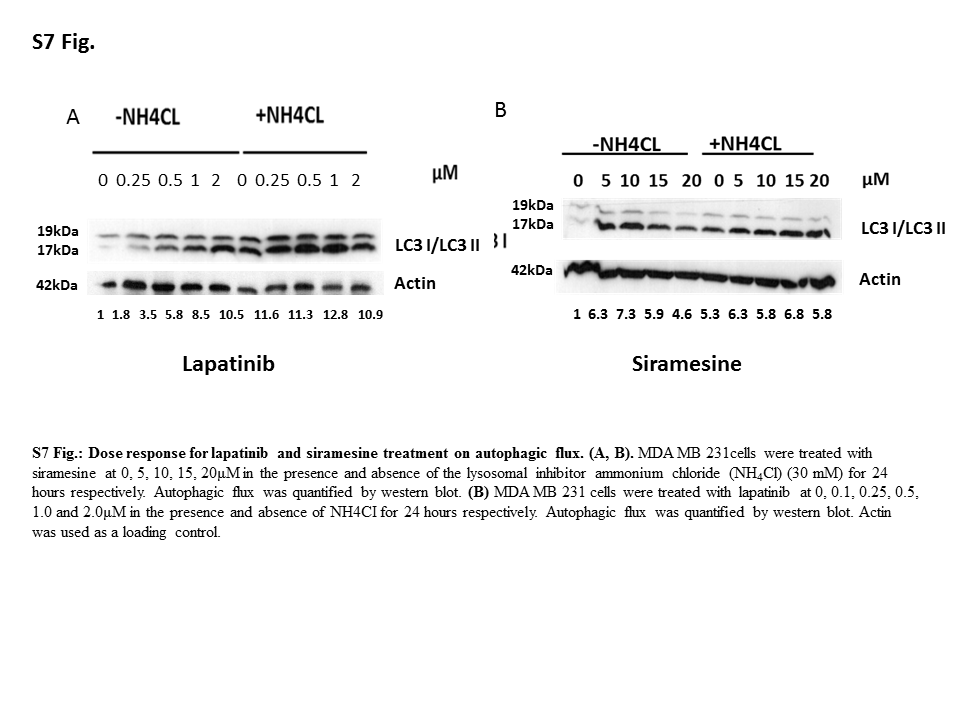

Supplement: S7 Fig — (A, B). MDA MB 231 cells were treated with siramesine at 0, 5, 10, 15, 20 microM in the presence and absence of the lysosomal inhibitor ammonium chloride (NH4Cl) (30 mM) for 24 hours respectively. Autophagic flux was quantified by western blot. (B) MDA MB 231 cells were treated with lapatinib at 0, 0.1, 0.25, 0.5, 1.0 and 2.0 microM in the presence and absence of NH4CI for 24 hours respectively. Autophagic flux was quantified by western blot. Actin was used as a loading control. (TIF) [file pone.0182921.s007.TIF]

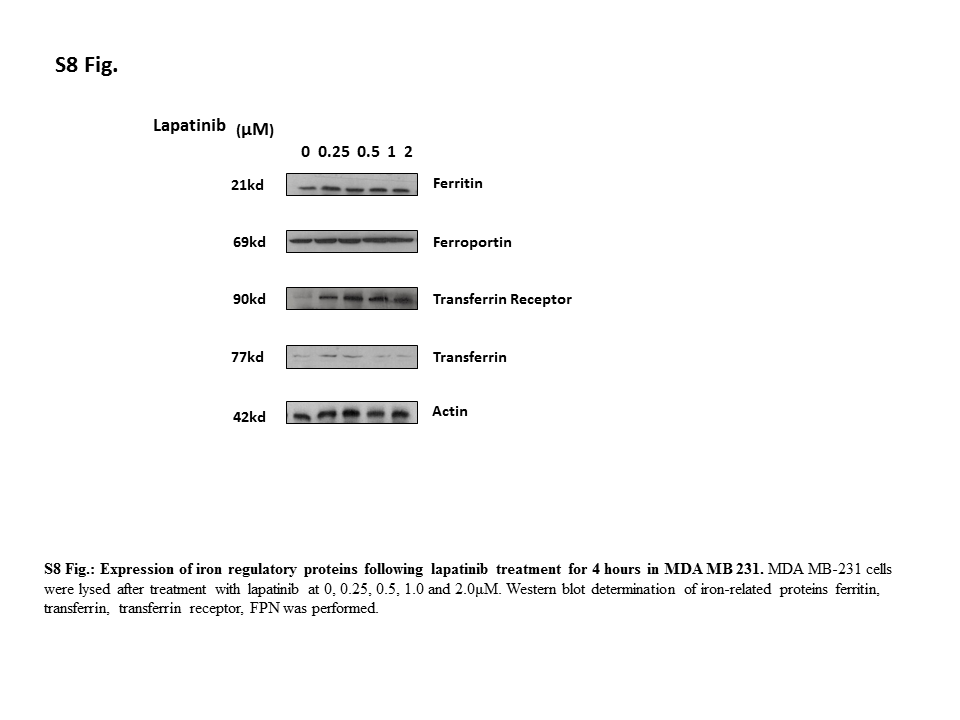

Supplement: S8 Fig — MDA MB-231 cells were lysed after treatment with lapatinib at 0, 0.25, 0.5, 1.0 and 2.0 microM. Western blot determination of iron-related proteins ferritin, transferrin, transferrin receptor, FPN was performed. (TIF) [file pone.0182921.s008.TIF]

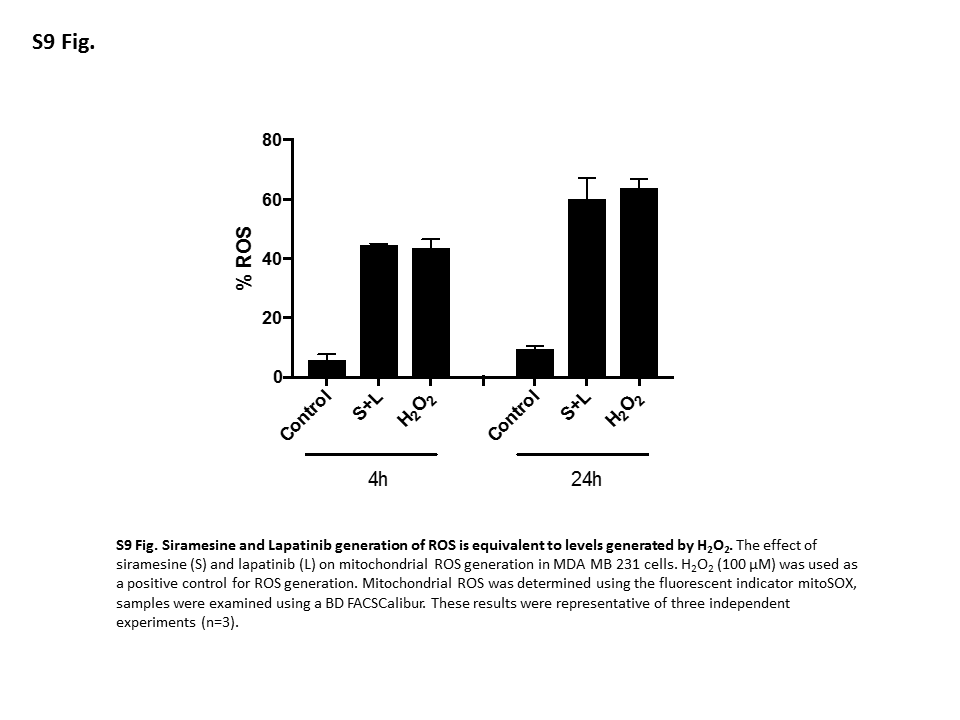

Supplement: S9 Fig — The effect of siramesine (S) and lapatinib (L) on mitochondrial ROS generation in MDA MB 231 cells. H2O2 (100 microM) was used as a positive control for ROS generation. Mitochondrial ROS was determined using the fluorescent indicator mitoSOX, samples were examined using a BD FACSCalibur. These results were representative of three independent experiments (n = 3). (TIF) [file pone.0182921.s009.TIF]

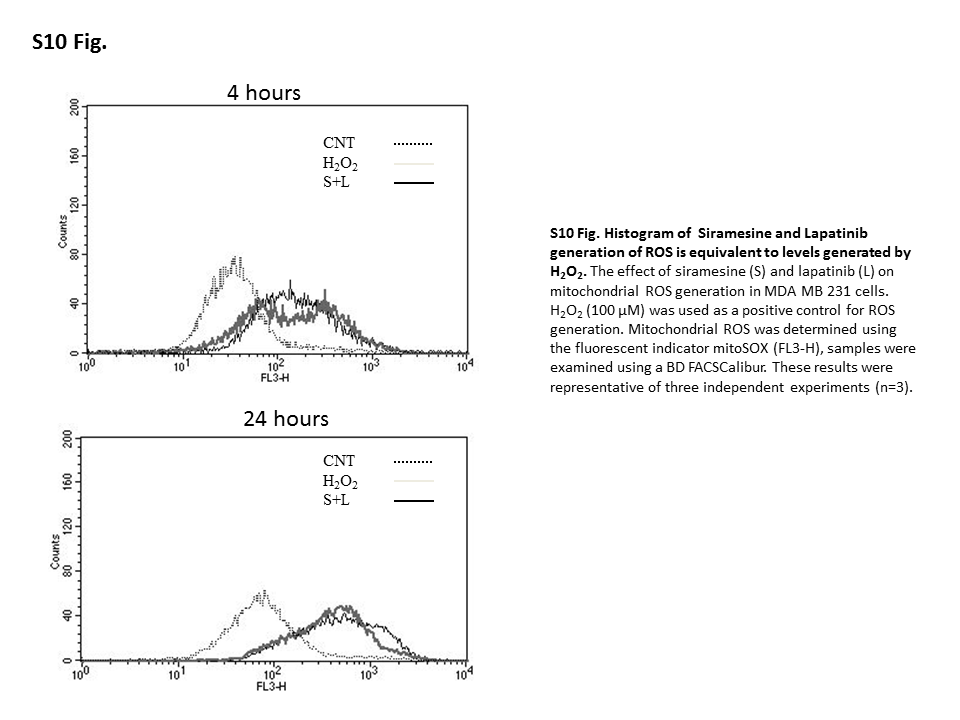

Supplement: S10 Fig — The effect of siramesine (S) and lapatinib (L) on mitochondrial ROS generation in MDA MB 231 cells. H2O2 (100 microM) was used as a positive control for ROS generation. Mitochondrial ROS was determined using the fluorescent indicator mitoSOX (FL3-H), samples were examined using a BD FACSCalibur. These results were representative of three independent experiments (n = 3). (TIF) [file pone.0182921.s010.TIF]

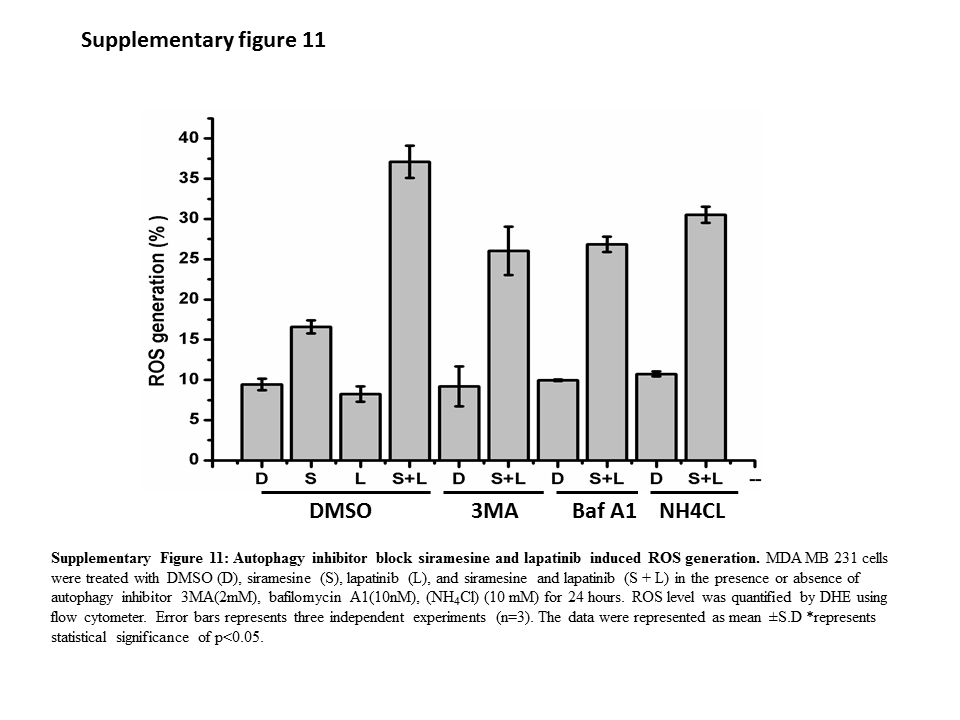

Supplement: S11 Fig — MDA MB 231 cells were treated with DMSO (D), siramesine (S), lapatinib (L), and siramesine and lapatinib (S + L) in the presence or absence of autophagy inhibitor 3MA (2mM), bafilomycin A1(10nM), (NH4Cl) (10 mM) for 24 hours. ROS level was quantified by DHE using flow cytometer. Error bars represents three independent experiments (n = 3). The data were represented as mean ±S.D *represents statistical significance of p<0.05. (TIF) [file pone.0182921.s011.TIF]

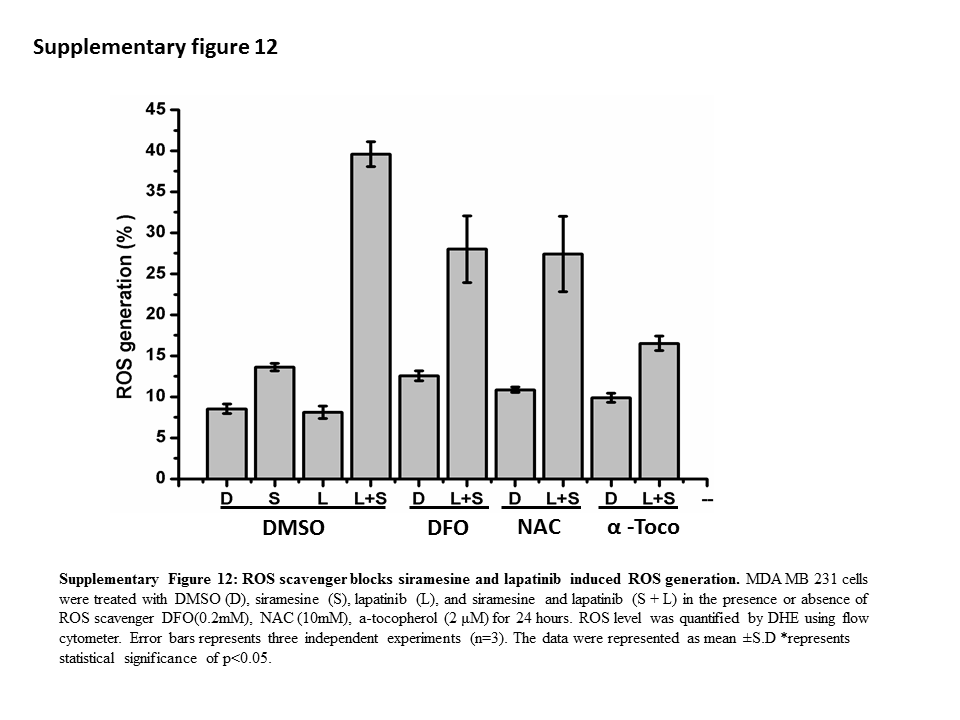

Supplement: S12 Fig — MDA MB 231 cells were treated with DMSO (D), siramesine (S), lapatinib (L), and siramesine and lapatinib (S + L) in the presence or absence of ROS scavenger DFO(0.2mM), NAC (10mM), a-tocopherol (2 microM) for 24 hours. ROS level was quantified by DHE using flow cytometer. Error bars represents three independent experiments (n = 3). The data were represented as mean ±S.D *represents statistical significance of p<0.05. (TIF) [file pone.0182921.s012.TIF]

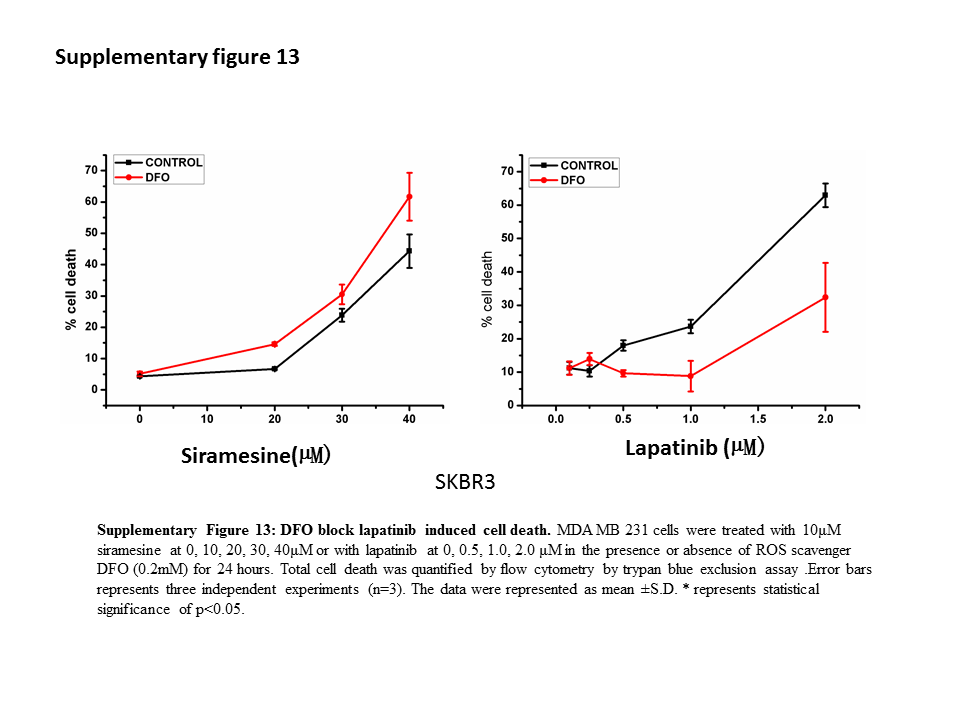

Supplement: S13 Fig — MDA MB 231 cells were treated with 10 microM siramesine at 0, 10, 20, 30, 40 microM or with lapatinib at 0, 0.5, 1.0, 2.0 microM in the presence or absence of ROS scavenger DFO (0.2mM) for 24 hours. Total cell death was quantified by flow cytometry by trypan blue exclusion assay. Error bars represents three independent experiments (n = 3). The data were represented as mean ±S.D. * represents statistical significance of p<0.05. (TIF) [file pone.0182921.s013.TIF]

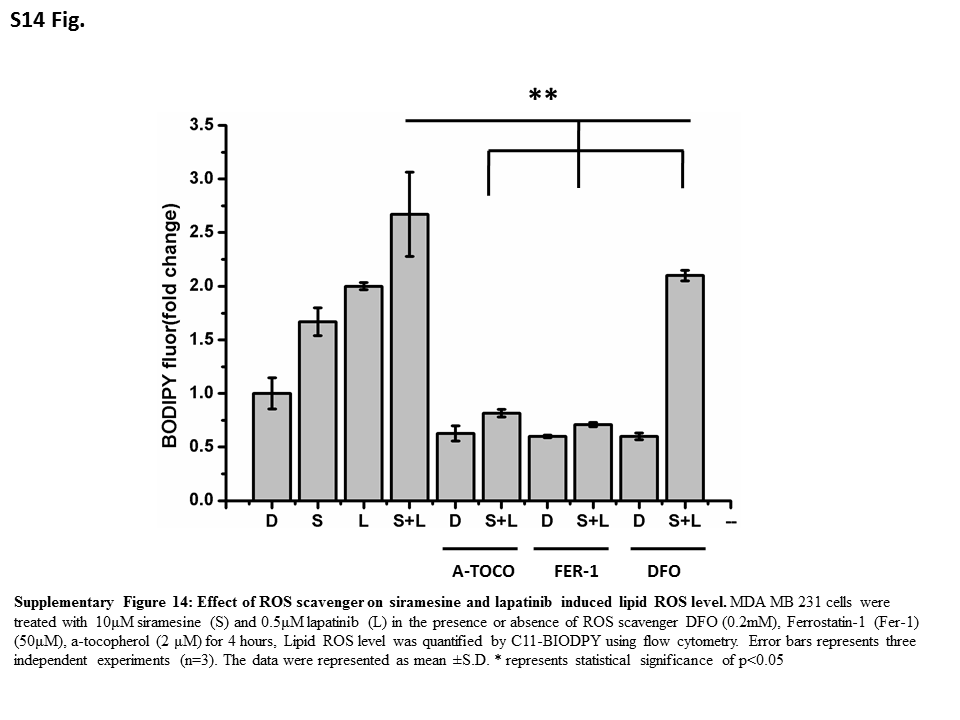

Supplement: S14 Fig — MDA MB 231 cells were treated with 10 microM siramesine (S) and 0.5 microM lapatinib (L) in the presence or absence of ROS scavenger DFO (0.2mM), Ferrostatin-1 (Fer-1) (50 microM), alpha-tocopherol (2 microM) for 4 hours, Lipid ROS level was quantified by C11-BIODPY using flow cytometry. Error bars represents three independent experiments (n = 3). The data were represented as mean ±S.D. * represents statistical significance of p<0.05. (TIF) [file pone.0182921.s014.TIF]

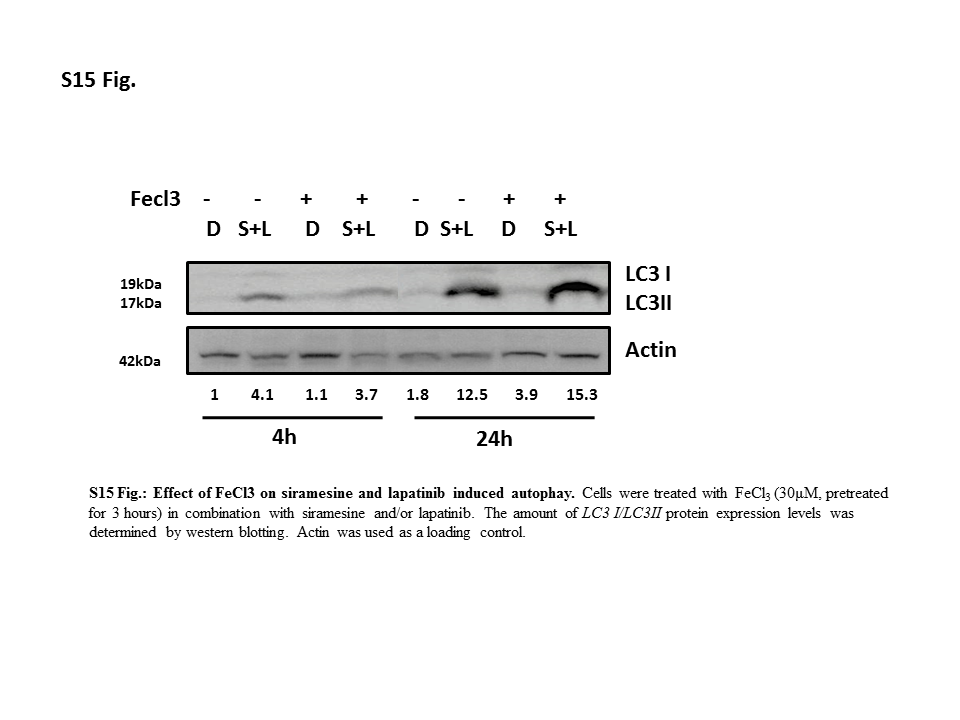

Supplement: S15 Fig — Cells were treated with FeCl3 (30 microM, pretreated for 3 hours) in combination with siramesine and/or lapatinib. The amount of LC3 I/LC3II protein expression levels was determined by western blotting. Actin was used as a loading control. (TIF) [file pone.0182921.s015.TIF]

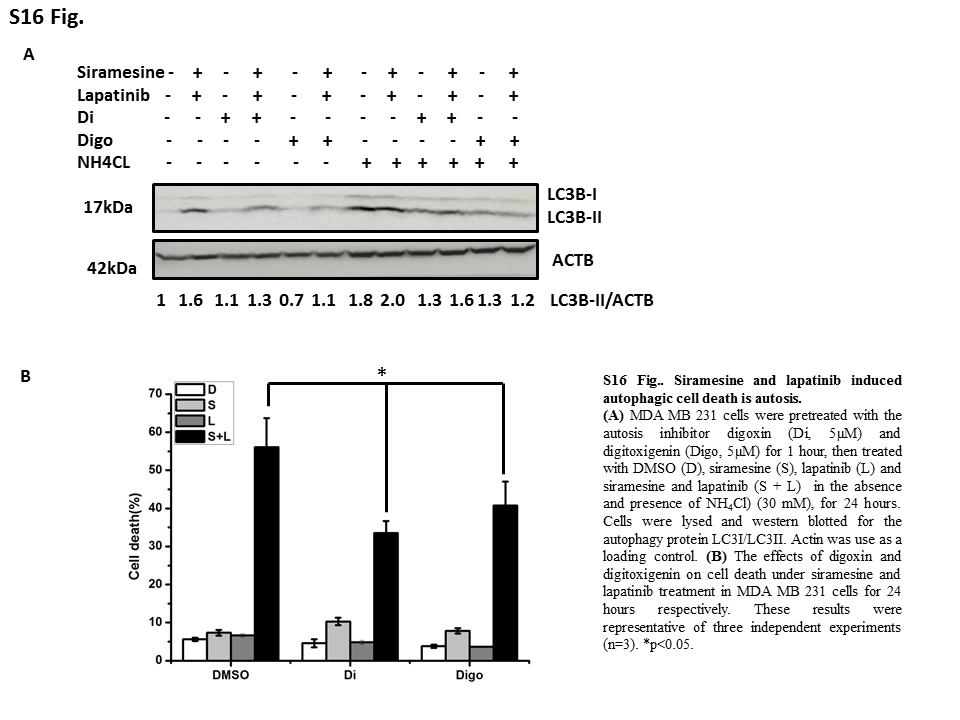

Supplement: S16 Fig — (A) MDA MB 231 cells were pretreated with the autosis inhibitor digoxin (Di, 5 microM) and digitoxigenin (Digo, 5 mciroM) for 1 hour, then treated with DMSO (D), siramesine (S), lapatinib (L) and siramesine and lapatinib (S + L) in the absence and presence of NH4Cl) (30 mM), for 24 hours. Cells were lysed and western blotted for the autophagy protein LC3I/LC3II. Actin was use as a loading control. (B) The effects of digoxin and digitoxigenin on cell death under siramesine and lapatinib treatment in MDA MB 231 cells for 24 hours respectively. These results were representative of three independent experiments (n = 3). *p<0.05. (TIF) [file pone.0182921.s016.TIF]

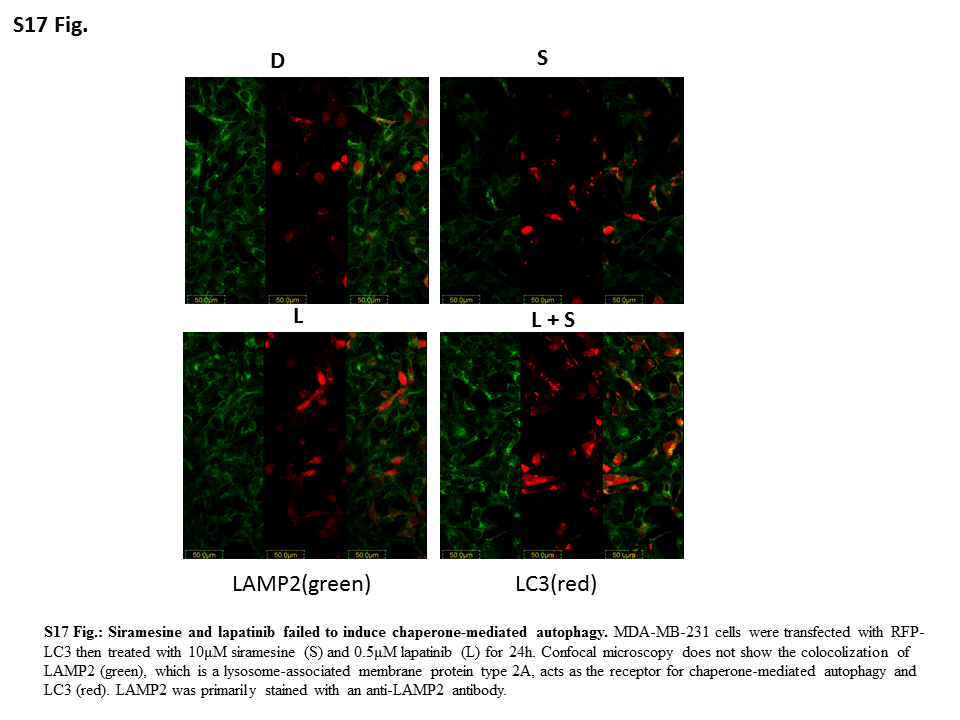

Supplement: S17 Fig — MDA-MB-231 cells were transfected with RFP-LC3 then treated with 10 microM siramesine (S) and 0.5 microM lapatinib (L) for 24h. Confocal microscopy does not show the colocolization of LAMP2 (green), which is a lysosome-associated membrane protein type 2A, acts as the receptor for chaperone-mediated autophagy and LC3 (red). LAMP2 was primarily stained with an anti-LAMP2 antibody. (TIF) [file pone.0182921.s017.TIF]
